# Differentiation of workers into soldiers is associated with a size reduction of higher-order brain centers in the neotropical termite *Procornitermes araujoi*

**DOI:** 10.1038/s41598-023-45221-0

**Published:** 2023-10-25

**Authors:** Lohan Valadares, Iago Bueno da Silva, Ana Maria Costa-Leonardo, Jean-Christophe Sandoz

**Affiliations:** 1 Evolution, Genomes, Behavior, and Ecology (EGCE), Université Paris-Saclay, CNRS, IRD, Gif-Sur-Yvette, France; 2 Laboratório de Cupins, Departamento de Biologia Geral e Aplicada, Instituto de Biociências, Universidade Estadual Paulista (UNESP), Rio Claro, SP Brazil

**Keywords:** Social behaviour, Animal behaviour

## Abstract

Comparing the size of functionally distinct brain regions across individuals with remarkable differences in sensory processing and cognitive demands provides important insights into the selective forces shaping animal nervous systems. We took advantage of the complex system of worker-to-soldier differentiation in the termitid *Procornitermes araujoi*, to investigate how a profound modification of body morphology followed by an irreversible shift in task performance are translated in terms of brain structure and size. This behavioural shift is characterised by a reduction of the once wide and complex behavioural repertoire of workers to one exclusively dedicated to nest defence (soldiers). In accordance with soldier’s reduced cognitive and sensory demands, we show here that differentiation of workers into soldiers is associated with a size reduction of the mushroom body (MB) compartments, higher-order brain regions responsible for multimodal processing and integration of sensory information, as well as learning, memory, and decision-making. Moreover, in soldiers, we found an apparent fusion of the medial and lateral MB calyces likely associated with its volume reduction. These results illustrate a functional neuroplasticity of the MB associated with division of labour, supporting the link between MB size and behavioural flexibility in social insect workers.

## Introduction

### Social insects: division of labour and neuroplasticity

Neuroecology theory posits that investment in neural tissue is selected to match the cognitive and/or sensory demands animals are confronted to^1^, implying that adult brain size may vary largely at both intraspecific and interspecific levels. Since an increase in brain size is associated with higher computational power, increased sensory processing capabilities and enlarged storage capacity^2^, a plausible advantage of a disproportionately larger brain might be an enhanced ability to learn new behaviours, to cope with novel or complex challenges in an ever-changing environment^3,4^. In bees, for instance, the probability of learning to associate a colour with a sugar reward is positively correlated with brain size, suggesting that large-brained species would be better learners than species with smaller brains^4^. Comparative analyses of the size of brains and brain compartments are thus useful for identifying adaptive patterns of neural investment^5^ and may provide important insights into the evolutionary forces shaping animal nervous systems.

Due to their elaborated division of labour, social insects (ants, termites, and some species of wasps and bees) are excellent models for testing neuroecological hypotheses. In these insects’ societies, only one or a few individuals undertake reproductive functions, while the remaining colony workforce is made up of non-reproductive individuals (the so-called workers and soldiers). These individuals engage in a wide range of tasks that are crucial to colony growth and maintenance, such as building, cleaning and repairing the nest, caring for the eggs and brood, foraging and ensuring colony defence, among others^6^. Task allocation within the colony is mostly based on age and/or body size differences among individuals^7^. This implies that workers and soldiers generally engage in different sets of tasks, with striking differences in their cognitive and sensory demands. Soldiers in general bear unique adaptive morphological features and, compared to workers, show a reduced behavioural repertoire strongly focussing on defence^7–12^. Note, however, that not all social insect species present a soldier caste. In hymenopteran societies (wasps, bees, and ants), soldiers comprise a subset of the worker caste and are only found in derived lineages of ants and stingless bee species^13–15^. In termites, however, workers and soldiers correspond to distinct castes emerging from complex developmental paths (detailed in the next section), and except for a few taxa in which they were lost, all termite species have a full soldier caste, with no equivalent in other social insects^16^.

The literature on brain plasticity underpinning division of labour in social hymenopterans is vast (bees^17–21^, ants^22–27^). Surprisingly, it is extremely scarce for one of the most speciose groups of social insects, the termites^28,29^. Overall, for protist-dependent termites (or also referred as to “lower termites”) and social hymenopterans, shifts in task performance are often associated with changes in the size of the mushroom bodies (MB), a brain region responsible for higher-order processing and integration of sensory information, as well as with learning and memory and decision making^17,18,20,21,23,28,29^. These findings generally indicate that a greater investment in the MB increases individual behavioural flexibility^23^, an essential feature of division of labour. Since eusociality in termites evolved independently from Hymenoptera^30^, investigating the link between neural investment and division of labour in termites offers an excellent opportunity for comparison with the better-known mechanism of neural investment in social hymenopterans. Both are eusocial but they show remarkable differences in ecological traits and caste developmental paths^30,31^.

### Castes in termites: development, behaviour, and brain size

Caste differentiation in termites takes place during postembryonic development, in which two major pathways, linear or bifurcated, occur depending on the species^32,33^. In the *linear* pathway, the colony workforce comprises the so-called pseudergates or “false” workers^34^, developmentally flexible immatures with the potential to differentiate into sterile (soldiers) and reproductive (alates and neotenics) castes. Such a caste development is only observed in protist-dependent termites with a one-piece nest life-style (termed ‘OP termites’). These termites use a single piece of wood as both shelter and food source, and therefore do not leave their nest^35^. In the *bifurcated* pathway, however, there is an early and irreversible bifurcation between the sterile and reproductive lineages^33^. A true sterile worker caste is observed in this system, which may further develop into a sterile soldier but is unable to differentiate into a winged reproductive individual^34^. This system is found in some protist-dependent termites and in all termitids (or also referred as to “higher termites”), with a multiple-piece nest life-style (‘MP termites’). In contrast to OP termites, foraging sites are separated from the nest of MP species, thus both workers and soldiers may explore the outside environment^35^.

**Figure 1:**
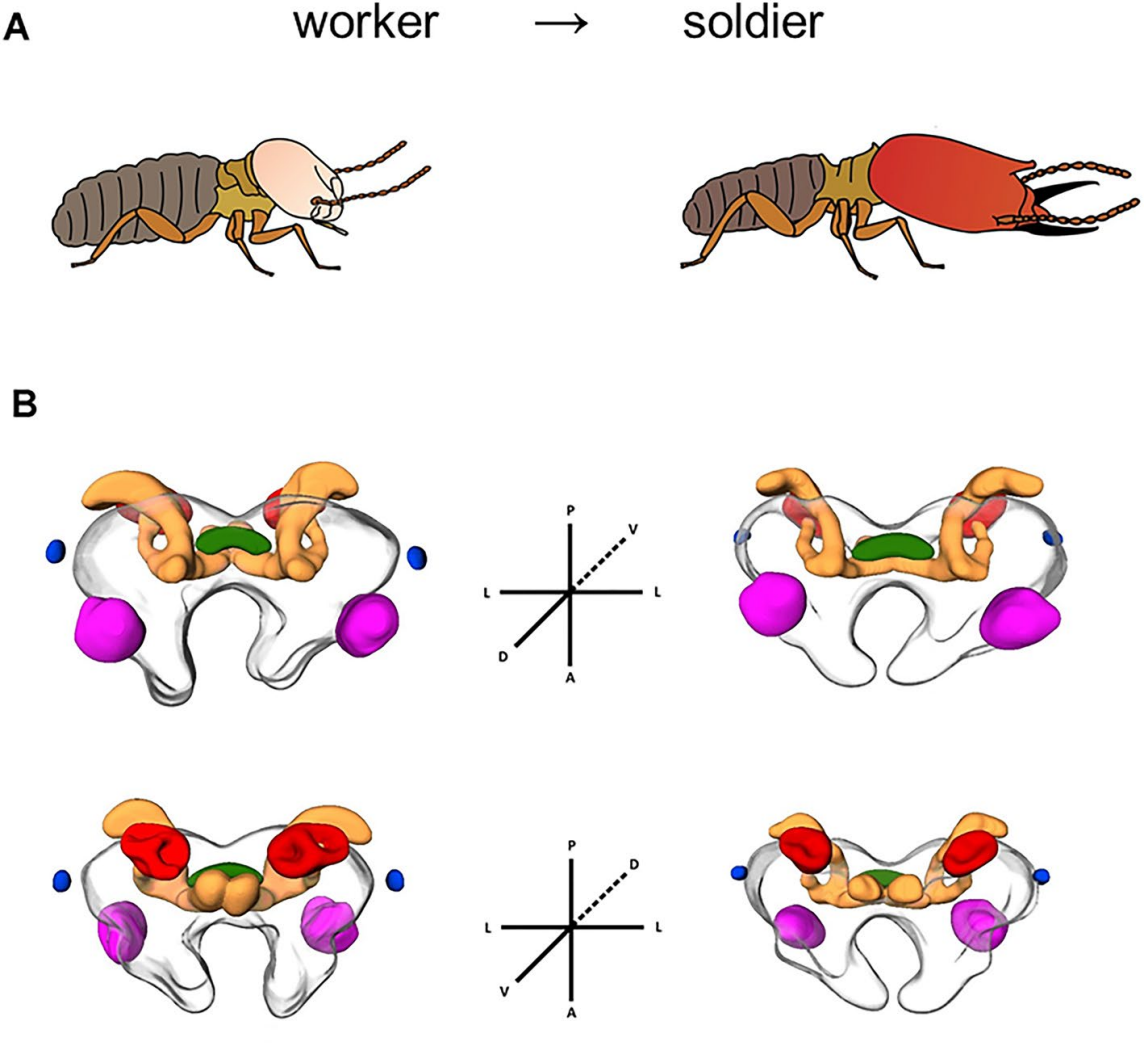
Body and brain morphology of sterile castes (workers and soldiers) of the higher neotropical termite *Procornitermes araujoi*. (**A**) Transition from worker to soldier is marked by an investment in body size traits adapted to nest defence (enlargement of the head capsule and development of weapons, such as the mandibles and exocrine glands). (**B**) 3-D modelling of functionally distinct brain regions of workers and soldiers: calyces (red), MB lobes (orange), antennal lobes (pink), central body (green), and the optic lobes (blue).

Termite soldiers differentiate from workers through two moults, going through a presoldier stage^36,37^. During this process, an intense morphogenesis takes place involving cell proliferation and degeneration according to the body part, triggered by hormones and a range of upregulated genes^38,39^. As a result, termite soldiers bear unique morphological and physiological adaptations to defend the colony (Fig. 1A), including enlarged and/or sharpened mandibles, a brush-like labrum, a frontal gland and the nasus, a horn-like frontal projection from which the defensive secretion is released^37^. The head morphology of termite soldiers is so profoundly modified for defence that these individuals depend on the workers for feeding and grooming^40^. Accordingly, the soldiers only perform defence tasks. Workers, on the contrary, perform a wide range of cognitively demanding tasks, such as foraging, building of nest structures, feeding of dependent castes, egg and brood care, corpse management, and grooming^41,42^. In addition, they also ensure passive defence by building and repairing the nest structure, and active defence by biting, grasping and, in some cases, performing suicidal behaviours against enemies^36,43–45^.

So far, studies of termite brain size were conducted on OP termites^28,29,46^ with a highly flexible caste development system, and very recently on a single subterranean MP species—that is, in this species termites forage in underground tunnels^47^. Therefore, the link between brain size and division of labour in termites remains largely unexplored, especially among MP species, which show true workers, and may explore the outside environment, and use different types of communication channels (e.g., mechanical and chemical signals). In this study we compared the volumes of a set of brain regions between workers and soldiers, two sterile castes of the Neotropical termite *Procornitermes araujoi* (Termitidae, MP termite). This species belongs to the subfamily Synterminitinae, endemic to the Neotropical region, in which soldiers possess both mechanical and chemical weapons associated with the head^48^. Furthermore, *P. araujoi* forage on the grounds around their nests, a stimulus-rich environment^88^. The development of powerful mandibles and the frontal gland reservoir in *P. araujoi* soldiers results in enlarged heads when compared to workers, turning this species into a suitable model for analysing brain changes accompanied by head modifications.

Here, we measured the volumes of regions associated with peripheral processing of olfactory and visual information, the antennal lobes (AL) and the optic lobes (OL) respectively, as well as the multimodal integration brain centres, the mushroom bodies (MB), in *P. araujoi* workers and soldiers. We also compared the volumes of the central body (CB), a brain region involved in orientation and navigation^49^. To estimate the overall investment of the two castes in sensory and integration brain regions, the sum of the five reconstructed neuropils was used. We tested the following hypotheses: (i) given the morphological, physiological, and behavioural changes occurring during worker-to-soldier differentiation in these termites^39^, we expected to find differences in the size of some brain regions between these castes; (ii) we also expected that a true worker caste, which performs a wider range of cognitively demanding tasks than soldiers, a caste restricted to defence, would invest more in high order processing neuropils (MB).

## Methodology

### Termite sampling

Apterous castes (workers and soldiers) of the termite *Procornitermes araujoi* Emerson 1952 were sampled at the São Paulo State University, in the city of Rio Claro, SP, Brazil (22°23′49.01″ S; 47°32′38.06″ W), during September 2022. One epigeal nest was fragmented with the aid of a pickaxe and carried to the laboratory where termites were carefully removed with forceps and placed on 9 cm Petri dishes filled with moistened filter paper. Revisions on the genus *Procornitermes* did not indicate the occurrence of polymorphism within workers and soldiers^50,51^. Thus, we consider individuals as monomorphic during our samplings. Four groups containing a suitable number of workers and soldiers were kept in plastic containers for brain dissections. Each container comprised moistened vermiculite, sugarcane bagasse as food source, and small pieces of the nest.

### Brain dissection and immunohistochemistry procedures

Termites were anaesthetised on ice and their head capsules were cut off and mounted in a petri dish containing a mix of paraffin and beeswax to prevent the head from moving. To estimate head volume, we measured head height and head width using a dissection stereomicroscope with an ocular calibrated with a 1-cm scale. Head height was measured from the distal point of the frontal pore at the tip of the nasus to the vertex, and head width was measured as the distance between the antennal sockets. We then applied the following formula of an ellipsoid^52^ to estimate head capsule volume for each termite subject, where head width and height are directly measured, but head depth is estimated by using half of head width as an approximation of head depth:

$${\text{Head}}\;{\text{capsule}}\;{\text{volume}} = {4}/{3} \times \pi \, \times \left( {1/2\;head\;width\;{\text{mm}}} \right) \times \left( {\;head\;height\;{\text{mm}}} \right) \times \left( {\;head\;depth\;{\text{mm}}} \right)$$

Termite brains were dissected in phosphate-buffered saline (PBS) solution. Using fine forceps and surgical scissors, the front of head capsule was carefully opened and the antennal nerves gently cut off. Then the brain was pulled out from the head capsule by grabbing it at the level of the subesophageal zone (SEZ). Given the small size of the brain and the hard-surrounding tissues, this procedure was necessary in termites, even though it caused some tissue perforations over the SEZ and the central brain region (protocerebrum). It is important to note that these small perforations did not alter the overall morphology of the protocerebrum, but prevented us from robustly measuring its volume. Also, pulling out the brain by grabbing it at the level of the SEZ preserved the entire morphology and volumetry of the brain neuropils of interest (Fig. 1B). Brains were fixed in ice-cold fixative solution (4% paraformaldehyde solution diluted in PBS pH ~ 7.3) for 2 h. They were washed in 0.2% PBST (PBS solution with 0.2% Triton X-100) for 24 h and blocked overnight in an antibody blocking solution (0.2% bovine serum albumin and 0.004% sodium azide (NaN_3_) diluted in PBST 0.2%). Brains were then incubated for 3 days in 1:30 primary antibody against synapsin (α-Synorf1, DSHB, USA). Synapsins are neuron-specific phosphoproteins, representing 9% of the total amount of all vesicle proteins in neurons wherein they play a critical role in the regulation of neurotransmitter release^53^. Because synapsins are abundant in pre-synaptic terminals, they have been widely used as a marker of synaptic active zones which allow visualization and separation of functionally distinct brain areas. The specificity of the antibody has been characterized in *Drosophila*^54^ and in the honey bee *Apis mellifera*^55^ and its affinity was confirmed in numerous neuroanatomical studies on diverse insect species^56–58^. After primary incubation, brains were again washed and incubated in 1:300 secondary antibody containing a fluorescent dye (Invitrogen Alexa Fluor 546, Fragment of Goat Anti-Mouse IgG, Thermo Fisher, EUA) for 3 days. Next, brains were dehydrated in ascending ethanol series (1 × 50%, 70%, 80%, 90%, 2 × 98%, 2 × 100%), placed in methyl salicylate as mounting medium for at least 48 h before confocal scanning, and were whole-mounted using aluminium slides covered by thin glass coverslips on both sides.

### Confocal microscopy and brain volumetry

We scanned whole-mounted brain samples using a laser scanning confocal microscope (LSM-700; Carl Zeiss, Jena, Germany). Optical sections (1024 × 1024 pixels) were acquired at 0.45 μm/pixel (x,y) with 1.31-μm intervals (z) using a W Plan-Apochromat 20 × /NA 1.0 water-immersion objective at 0.5 × digital zoom. For reconstruction of brain neuropils, we imported confocal image stacks into the three-dimensional (3D) analysis software Amira (version 5.4.3; FEI, Berlin, Germany). For extracting neuropil volumes, the brush tool was used to outline manually and individually the following neuropils: the antennal lobes (AL), the central body (CB), the optic lobes (OL), and the compartments of the mushroom body (MB): calyces, vertical lobe, medial lobe, and peduncle (Fig. 1B). The sum of the volumes of the five neuropils was used as a proxy to estimate the volume of the “sensory and integration brain region”. As we explained, the central brain region (protocerebrum) is fused to the SEZ and was deformed during dissections in most samples, precluding its volumetric measure. Thus, we could only reconstruct this region in 3 workers and 6 soldiers. Although synapsin staining allowed us to reconstruct the medial lobe, the vertical lobe and the peduncles, we were not able to precisely separate their intersection borders and thus, MB lobes were considered as a single unit for analysis. Also, synapsin staining did not allow counting individual glomeruli in the termite AL, and we thus only considered the whole AL volume. The sum of the five reconstructed neuropils was used to measure the overall investment in sensory and integration neuropils.

By comparing the sum of the five reconstructed neuropils between termite castes, we were able to test whether the differentiation of workers into soldiers affects the size of the sensory and integration brain region. In case a significant effect was detected, we then tested separately whether such difference emerge from differences at the level of specific neuropils—that is, if one or few neuropils are affecting the size of the sensory and integration brain region—or if all neuropils contribute similarly to the size difference between the two phenotypes.

### Statistical analyses

Because the remaining brain region (protocerebrum + SEZ) could only be reconstructed in 9 individuals out of 20 (see “Methodology”), we did not include this region in the statistical analyses. We used the volume of the head, the volume of each reconstructed brain neuropil, as well as the sum of the five reconstructed neuropils (sensory and integration brain region) as response variables. We checked the normality of data distribution using the Shapiro–Wilk’s test. Since all variables showed non-normal distributions, we compared neuropil sizes between workers and soldiers using the Mann–Whitney U test. Regarding the allometric scaling analysis between head volume and the volume of sensory and integration brain region, we used least-square means regression (OLS) on log-transformed values to estimate *a* and *b* in the scaling equation *y* = *aM*^*b*^*, as log*_*10*_*(y)* = *log*_*10*_* (a)* + *b* × *log*_*10*_* (M)*. To test the null hypothesis (H_0_) of isometry, a separate linear model was calculated and tested against *b* = 1 using a two-tailed *t* test^59^. All statistical analyses were done in R 4.0.3^60^.

## Results

### Termite brain morphology

**Figure 2:**
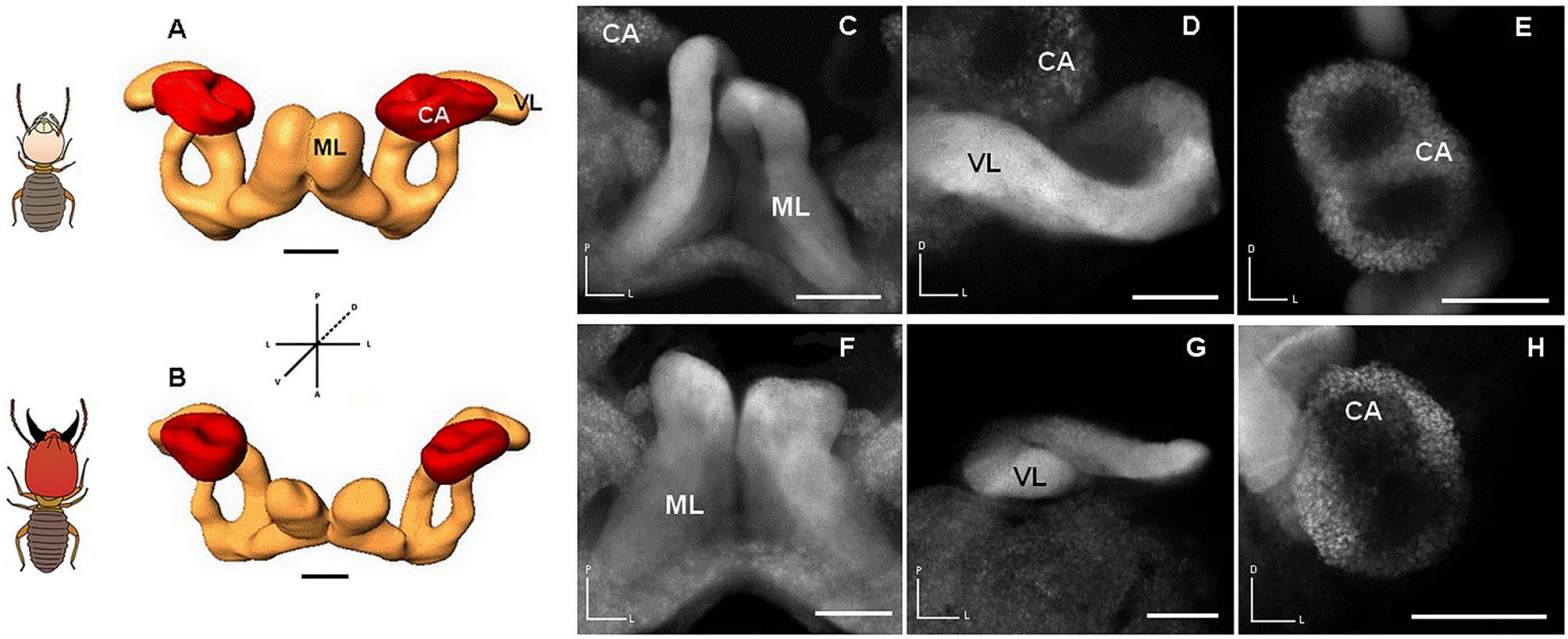
Morphology of the MB compartments between workers and soldiers of *P. araujoi*. (**A**,**B**) 3-D modelling of MB compartments in workers and soldiers, respectively. (**C**–**H**) Synapsin staining of the MB compartments revealed by laser scanning confocal microscopy: medial lobes (ML), vertical lobes (VL), and calyces (CA). Scale bar: 100 μm.

The brain morphology of *P. araujoi* is characterised by highly elaborated mushroom body lobes (Fig. 2) that together represented 51.5% of the reconstructed neuropils (data pooled for both soldiers and workers). Both medial and vertical lobes were greatly elongated into loop structures (Fig. 2A–D,F,G). The MB calyces (a pair on each side) represented 13% of the reconstructed neuropils, and appeared to be nearly fused into a single structure (Fig. 2E,H). In soldiers, the looping of the lobes was less obvious (Fig. 2F,G), and the fusion of the MB calyces was more evident (Fig. 2H) possibly due to a reduction in MB size, as detailed below. In accordance with the eyeless condition of both castes, their OL were remarkably small (making up less than 1% of the reconstructed neuropils) and packed into a roundish structure in which the subcompartments (the lamina, the medulla, and the lobula) could not be distinguished (Fig. 1B). The AL and the CB comprised, respectively, 31.6% and 2.8% of the reconstructed neuropils.

### Comparison of neuropil size between workers and soldiers

**Figure 3:**
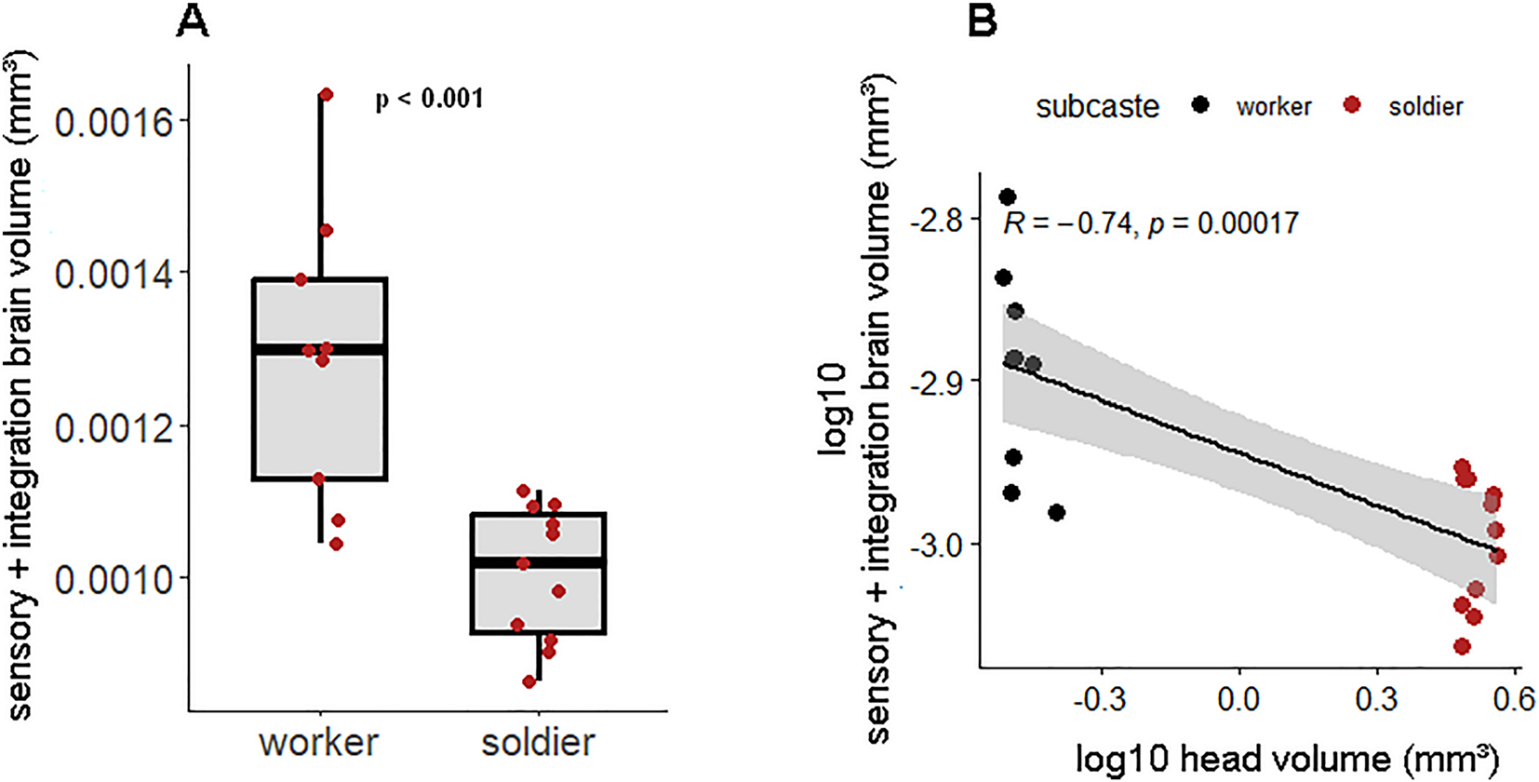
Differences in the size of the sensory and integration brain region between the sterile phenotypes (workers and soldiers) of the termite *P. araujoi*. (**A**) Boxplot showing overall size of sensory and integration neuropils (sum of five reconstructed neuropils) between workers and soldiers. (**B**) Relationship between the volume of the sensory and integration brain region (sum of the volume of the five chosen neuropils) and head volume across *P. araujoi* sterile phenotypes. Indicated *p* values represent a significant outcome after a Mann–Whitney U test (**A**) and least-square means regression (**B**).

The volume of the sensory and integration brain region (represented by the sum of the five reconstructed neuropils) was on average 24% smaller in soldiers than in workers (Fig. 3A) (workers, median: 0.0013, Q1: 0.0011—Q3: 0.0014 mm^3^; soldiers, median: 0.00101, Q1: 0.0009—Q3: 0.0011 mm^3^). This difference is highly significant (Mann–Whitney Test; U = 91, p < 0.001). Conversely, the volume of the head capsule was 10 times larger in soldiers than in workers (mean ± SD, worker: 0.332 ± 0.027 mm^3^; soldier: 3.288 ± 0.245 mm^3^), this difference is significant (Mann–Whitney Test; U = 8, p < 0.001). This suggests that the differentiation of workers into soldiers is associated with an enlargement of the head capsule combined with a size reduction of the sensory and integration brain region. Indeed, our allometric analyses showed a hypoallometric relationship (or negative allometry) between head volume and the sum of the five reconstructed neuropils (Fig. 3B), characterised by a slope < 1 which significantly differed from isometry (b = 1) (*b* = − 0.74, r^2^ = 0.547, 95% CI − 0.171 to − 0.059, *t*(18) = 18.55, p < 0.001).

**Figure 4:**
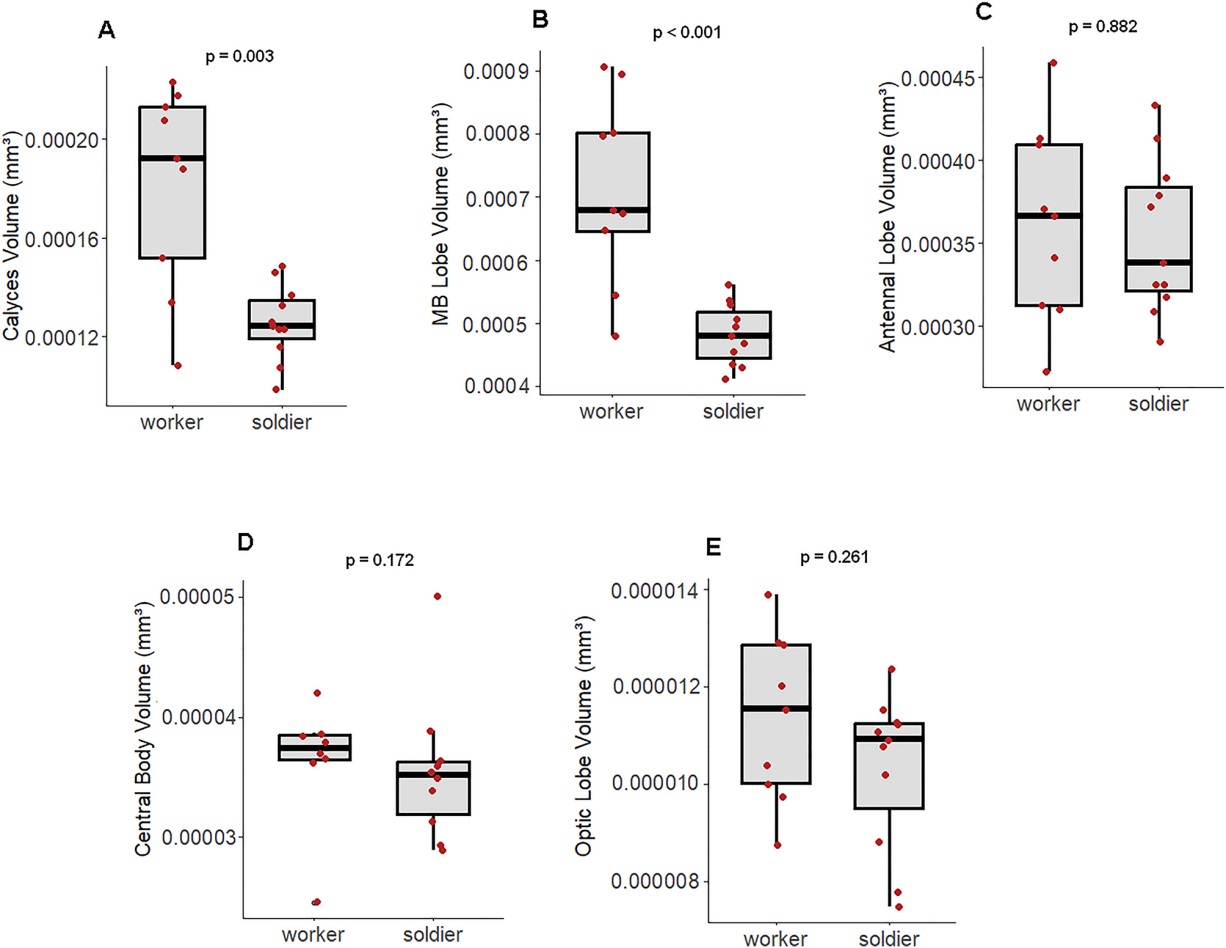
Boxplots showing the volume (mm^3^) of reconstructed neuropils between workers and soldiers of the neotropical termite *P. araujoi*. Indicated *p* values represent a significant outcome after a Mann–Whitney U test.

We next asked whether the brain size reduction observed for soldiers is accompanied by differential investment in specific brain region(s). We found that, in soldiers, the absolute volumes of the MB compartments (calyces and lobes) were significantly smaller than in workers (Fig. 4A,B) (calyces, *U* = 87, p = 0.003; MB lobes, *U* = 93, p < 0.001). The other reconstructed neuropils, the AL, the OL and the CB, did not differ in volume between workers and soldiers (Fig. 4C–E) (AL, *U* = 52, p = 0.882; OL, *U* = 65, p = 0.261, CB, *U* = 56, p = 0.172). These results indicate that differentiation of workers into soldiers is associated with a reduced investment in brain structures associated with higher-order processing and integration of sensory information, as well as with learning and memory. Since we could not reconstruct the remaining brain region (protocerebrum + SEZ) for most samples (see “Methodology”), we calculated its average size based on the few available samples (3 workers and 6 soldiers) and present this result in the supplementary material of this article (see Suppl. File 1). The reconstruction of the remaining brain region in this small group of samples allowed us to estimate the total volume of the brain (5 chosen neuropils + protocerebrum + SEZ), and consequently, to estimate the proportion that is occupied by each neuropil in the brain by obtaining the ratio between neuropil volume and total brain volume. Due to our low sample size, one should be careful when drawing conclusions about relative measures, however, these data suggest that workers may invest more in sensory + integration neuropils while soldiers had a higher investment in the remaining brain region (Supplementary Fig. 1).

## Discussion

In this work, we investigated how a profound modification in body morphology followed by an irreversible shift in task performance are translated in terms of neural investment in sensory and integration brain neuropils. We took advantage of the complex system of worker-to-soldier differentiation in termites, which is marked by an astonishing investment in body size traits adapted to colony defence. This behavioural shift is characterised by a drastic reduction of the once wide and highly flexible behavioural repertoire of workers to one exclusively dedicated to defence (soldiers). In accordance with the worker status for higher cognitive and sensory demands, we report here that the sensory and integration brain region of workers is larger than that of soldiers due to a size reduction of the MB in soldiers, all other measured brain centers maintaining the same volume (Fig. 4). The MB is a higher-order brain neuropil responsible for multimodal processing and integration of sensory information, as well as for learning and memory^20,61,62^. In social insects, a large amount of literature shows that shifts in task allocation underlying division of labour are intimately associated with plasticity at the level of the MBs^17,18,20,21,23^. For instance, workers of the ant *Camponotus floridanus* engaged in foraging show a greater investment in the MB than their nursing nestmates, while idle sisters have a significantly smaller MB than the former groups^63^. A similar growth of the MB is observed when bees change from nursing to foraging tasks, suggesting that the experience outside the colony causes neuropil growth^18,19^.

Our results obtained here for *P. araujoi*, which show striking differences in terms of social organisation compared to all other eusocial species in which brain size and division of labour were studied (Hymenoptera, exclusively), provide more support to the idea that investment in the MB promotes behavioural flexibility, a key feature underlying division of labour. According to Farris & Strausfeld^61^, the first and only study to date to provide a detailed description of MB morphology in termites, the elongation and looping of the vertical and medial lobes appear to be a derived trait found only in Termitidae^61^. As we report here, the brain morphology of *P. araujoi* is also characterised by elaborated MB lobes (Fig. 2), a region known for its role in the modulation of behavioural activity, formation, storage, and retrieval of memories, and learning^62–67^. Thus, elaboration of the MB lobes suggests an adaptation to the MP lifestyle of termitids, which in contrast to OP termites, may forage outside the nest (a rich sensory environment) and communicate using a complex system of vibratory and chemical signals. Interestingly, in soldiers of *P. araujoi*, the looping of vertical and medial lobes is far less evident, and the fusion of the medial and lateral calyx more marked (Fig. 2F–H), supporting the idea of a reduced investment in these structures.

The differentiation of termite workers into soldiers is marked by several morphogenetic and behavioural changes, including the enlargement of the head and the development of weapons, such as the mandibles and/or glands^39^. Given the hypoallometric relationship between the volume of the sensory and integration neuropils and head volume (Fig. 3B), we presume that such an enlargement of the head capsule of *P. araujoi* soldiers is related to (i) the anchoring of the powerful mandibular muscles, which are much more developed in termite soldiers^68,69^, as well as (ii) harbouring the frontal gland and its reservoir^70^. Interestingly, in several termite species, it has been reported that worker-to-soldier differentiation is associated with an enlargement of the subesophageal zone (SEZ), due to an increase in size of the mandibular motor neurons associated with mandibular movements^46^. The enlargement of the SEZ in soldiers seems to be associated with their behavioural specialisation to defence by increasing bite efficiency^46^. Indeed, based on the few samples in which we could reconstruct the total brain size (SEZ + protocerebrum + sensory and integration neuropils), we observed that in soldiers the remaining brain region (SEZ + protocerebrum) is larger. Thus, at the level of the overall brain size, a higher investment in the remaining brain region in soldiers seems to counterbalance their loss of investment in sensory and integration neuropils—which means that, in the sampled species, the overall size of the brain may not differ between workers and soldiers (Supplementary Table 02). This possible scenario is supported by the results obtained for another MP termite species, in which cross-sectional (two-dimension) area measurements of overall brain size did not indicate differences between workers and soldiers^47^.

It is important to note that worker-to-soldier differentiation in termites goes through an intermediate stage (termed presoldier), whose behavioural function is not well understood, although it is thought to be an important step during the morphogenesis of the soldier^39,71^. Brain size and neuropil investment in presoldiers remains to be explored. Based on the observed differences between workers and soldiers, it is possible to infer that brain anatomy in *P. araujoi* does not undergo any drastic change and/or rearrangement in the presoldier stage, contrary to head muscles^71^. Different from holometabolous insects, in which the larval brain is simplified to accommodate the sensory and motor demands of the larva^72,73^, it seems that for insects with direct development, brain morphology is developed early and, compared to muscle development, quite preserved across different stages^73^. Previous studies on brain development in locusts, another group of hemimetabolous insects, have shown that direct development results in a hatchling possessing an essentially mature, but miniature brain, which is used for both the nymphal and adult stages^73,74^. Also, the only consistent change in brain morphology throughout locust postlarval development is the increase of somata size in particular sets of neurons^73^.

Based on our results, neuropil size differences reported between workers and soldiers may emerge from (i) ontogeny, constituting an intrinsic result of the worker-to-soldier moulting, and detected, for instance, in presoldiers and/or recently-emerged soldiers, or (ii) experience, if neuropil size reduction occurs later in the lifespan of soldiers, thus reflecting their highly specialized status, with less demands for sensory processing and cognitive challenges. It could also be a combination of both ontogeny and life experience affecting MB size in the sampled species. It is interesting to note that MB volume varied greatly among workers (Fig. 4A,B). Since we could not distinguish worker groups due to their similar size, it is possible that we sampled different-aged individuals. Task allocation in termites relies, among other factors, on age^41,75^, which may result in changes of the MB volume as a response to differences in experience related to age polyethism.

The volume of the other reconstructed neuropils (AL, OL, and the CB) did not differ between workers and soldiers of *P. araujoi* (Fig. 4C–E). The AL acts as the primary olfactory brain region, receiving input from sensory neurons in the antennae and processing information concerning pheromones and environmental odours^76^. Since eusocial insects in general are primarily olfactory-oriented individuals, and *P. araujoi* workers and soldiers show striking differences in their behavioural repertoires, we initially expected to find volumetric differences between the ALs of these termite castes, based on the premise that olfactory demand would differ between them. Termite social interactions are mediated by both mechanical and chemical cues. The latter include cuticular hydrocarbons, which act as social recognition pheromones, as well as alarm and trail pheromones secreted by the frontal and sternal glands, respectively^60,77^. The emergence of alarm pheromones is suggested to have occurred only within MP termite species, which may face different challenges outside the nest and use these volatiles to alert nestmates^78^. Moreover, it has been shown that the secretion from the frontal gland of *P. araujoi* soldiers triggers an alarm response from conspecifics and is equally perceived by other soldiers and workers, which become aggressive^79^. Thus, the results obtained in this work either reflect that the gross volume of the AL is not a good predictor for differences in olfactory demand, or that *P. araujoi* castes do not differ in their olfactory demand, which is reflected by similar investment in AL size (Fig. 4C). Further studies on pheromonal perception and modulation in termites, as well as detailed morphological studies of AL glomeruli (AL functional units), may shed light on the link between olfactory demand and AL morphology.

Sterile castes of termites are usually blind, thus only individuals of the imaginal line show a gradual differentiation of the OL^28,33^, reaching the highest development among alates, who use vision during dispersal and for finding potential mating partners^42,47,80^. In termites, the development of eyes is probably linked to that of other imaginal traits such as gonads and wings^81,82^. Within sterile castes and neotenic reproductives, the development of these traits is fully or partially arrested during post-embryonic development^81,82^. Thus, imaginal features, including the eyes, are absent in workers and soldiers, which show only primordia of eyes, characterised by undifferentiated cell masses on the epidermis, but without any subsequent development^83, 84^. Within neotenics, the expression of eyes, wings, and gonads is under modular and heterochronic regulation, resulting in mosaic-like phenotypes^81,82,85–87^. As expected for *P. araujoi* workers and soldiers, OL volume was extremely low (around 1% of the reconstructed neuropils) and did not differ between them*,* which therefore agrees with the eyeless condition of these castes. For other social insects, behavioural specialisation clearly reflects on visual system phenotypes. For instance, hovering bees of *Tetragonisca angustula* are engaged in the recognition and interception of heterospecific bees, tasks relying mostly on vision, and a higher investment in the OL has been reported for these individuals^20,21^. In leafcutter ants, individuals that perform tasks inside the lightless galleries and outside of the nest also show different investments in the OL^26^.

We conclude that, transitioning from a broad, highly flexible behavioural status (workers) to a more specialised one promoting efficiency to a particular function (defence by soldiers), is associated in *P. araujoi* with a decrease in the size of higher-order brain regions (the MB) intimately associated with learning, memory, and decision-making. These results illustrate a functional neuroplasticity of the MB associated with division of labour, supporting the link between MB size and behavioural flexibility in social insect workers.

### Supplementary Information

Supplementary Information 1.

Supplementary Information 2.

Supplementary Information 3.

## Data Availability

The data supporting the findings of this study are available in the Supplementary Material of this article.
