# Supplementary material for: Differentiation of workers into soldiers is associated with a size reduction of higher-order brain centers in the neotropical termite Procornitermes araujoi
Source: Sci Rep. 2023 Oct 25;13:18279. doi: 10.1038/s41598-023-45221-0 (PMC10600217; doi:10.1038/s41598-023-45221-0)

**SUPPLEMENTARY FILE**

**Differentiation of workers into soldiers is associated with a size reduction of higher-order brain centers in the neotropical termite *Procornitermes araujoi***

Lohan Valadares, Iago Bueno da Silva, Ana Maria Costa-Leonardo, Jean-Christophe Sandoz

--

Supplementary Table 01 – absolute and relative (neuropil volume divided by sensory and integration brain area) volumes of brain neuropils, in workers and soldiers of the Neotropical termite *Procornitermes araujoi*.

|  | **Worker (n=9)** | | **Soldier (n=11)** | |
| --- | --- | --- | --- | --- |
|  | absolute (mm^3^) | relative | absolute (mm^3^) | relative |
| MB calyces | 0.00018 | 0.14 | 0.00012 | 0.12 |
| MB lobes | 0.00071 | 0.55 | 0.00048 | 0.48 |
| Antennal lobe | 0.00036 | 0.28 | 0.00035 | 0.35 |
| Optic lobe | 0.00001 | 0.008 | 0.00001 | 0.01 |
| Central body | 0.00003 | 0.022 | 0.00003 | 0.04 |
| Sensory + integration brain region ^a^ | 0.00130 |  | 0.00099 |  |

^a^ sum of the five reconstructed brain neuropils

Supplementary Table 02 – absolute and relative (neuropil volume divided by total brain volume) volumes of brain neuropils, in 3 workers and 6 soldiers of the Neotropical termite *Procornitermes araujoi*.

|  | **Worker (n=3)** | | **Soldier (n=6)** | |
| --- | --- | --- | --- | --- |
|  | absolute (mm^3^) | relative | absolute (mm^3^) | relative |
| MB calyces | 0.00018 | 0.055 | 0.00012 | 0.035 |
| MB lobes | 0.00074 | 0.223 | 0.00048 | 0.143 |
| Antennal lobe | 0.00039 | 0.12 | 0.00036 | 0.11 |
| Optic lobe | 0.00001 | 0.003 | 0.00001 | 0.002 |
| Central body | 0.00003 | 0.009 | 0.00003 | 0.008 |
| Protocerebrum + SEZ | 0.00191 | 0.59 | 0.00234 | 0.702 |
| Total brain | 0.00326 |  | 0.00334 |  |

Supplementary Figure 1 – relative size (ratio between neuropil volume and total brain volume) of each reconstructed brain region (MB calyces, MB lobes, antennal lobe, optic lobe, central body, and remaining brain area (protocerebrum + SEZ)) in 3 workers and 6 soldiers of the Neotropical termite *Procornitermes araujoi*


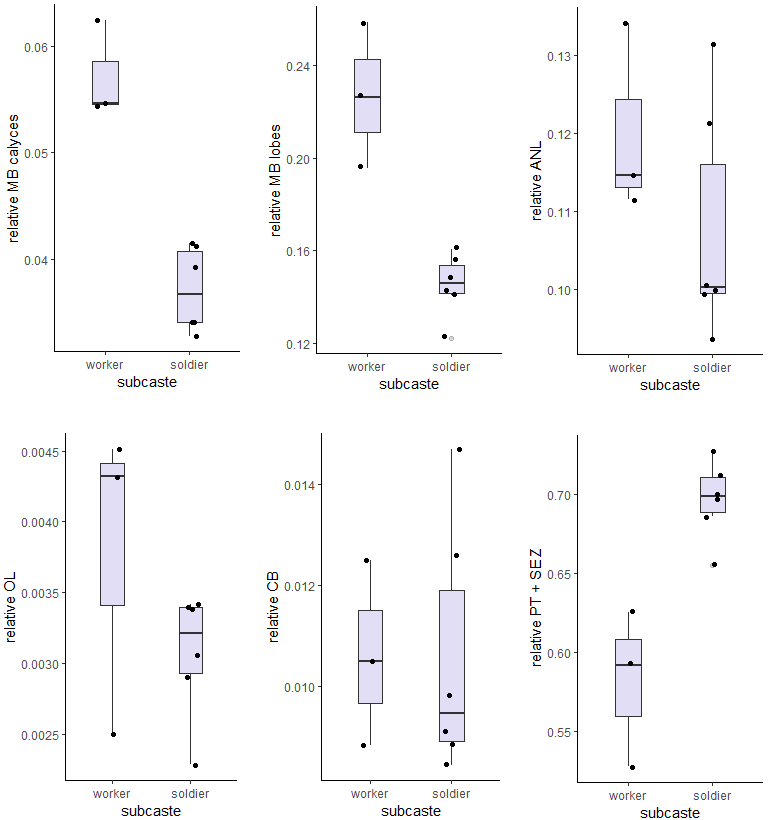

Supplement: Supplementary file 2 — Supplementary Information 2. [file 41598_2023_45221_MOESM2_ESM.docx]
